# Effects of Dietary Cottonseed Oil and Cottonseed Meal Supplementation on Liver Lipid Content, Fatty Acid Profile and Hepatic Function in Laying Hens

**DOI:** 10.3390/ani11010078

**Published:** 2021-01-04

**Authors:** Ao Yang, Cong Zhang, Beiyu Zhang, Zhiyun Wang, Luoyi Zhu, Yang Mu, Shuai Wang, Desheng Qi

**Affiliations:** Department of Animal Nutrition and Feed Science, College of Animal Science and Technology, Huazhong Agricultural University, Wuhan 430070, China; yangao@webmail.hzau.edu.cn (A.Y.); zhangcong@webmail.hzau.edu.cn (C.Z.); zhangbeiyu@webmail.hzau.edu.cn (B.Z.); zhiyun_wang_hzau@163.com (Z.W.); 11917023@zju.edu.cn (L.Z.); 20140017@hbut.edu.cn (Y.M.); wangshuai@mail.hzau.edu.cn (S.W.)

**Keywords:** cottonseed oil, cottonseed meal, cyclopropenoid fatty acids, free gossypol, lipid metabolism, serum biochemistry

## Abstract

**Simple Summary:**

Cottonseed by-products have been considered for use as nutrients in animal diets for a long time. However, the antinutrients, such as free gossypol and cyclopropene fatty acids, from cottonseed have caused numerous adverse effects on animal production. Commonly studies were concentrated on the toxicity of free gossypol, while the toxicity of cyclopropene fatty acids in cottonseed oil was neglected. The current study showed that dietary supplementation of degossypolized cottonseed oil containing cyclopropene fatty acids in diets contributed to dramatic changes in the fatty acid profile in tissues and elevated serum cholesterol level in hens. Therefore, it should raise public concerns about the application of cottonseed oil in both animal and human diets because of its long tradition of use in human food processing.

**Abstract:**

Antinutrients, such as cyclopropene fatty acids (CPFAs) and free gossypol (FG), present together in cottonseed have caused numerous adverse effects on liver health and egg quality of laying hens, which are both likely to be related to a disturbance in lipid metabolism. This experiment employed a 3 × 3 factorial arrangement using corn–soybean-meal-based diets supplemented with different levels of cottonseed oil (0%, 2%, or 4% CSO) containing CPFAs and cottonseed meal (0%, 6%, or 12% CSM) containing FG to elucidate the effects of them or their interaction on fatty acid profile, lipid content, and liver health of laying hens. An overall increase in fatty acid saturation and an overall significant decrease (*p* < 0.05) in monounsaturated fatty acids (MUFAs) were shown in the livers of hens fed diets with either 2% or 4% CSO. Meanwhile, the concentration of liver cholesterol, serum cholesterol, and serum LDL-c of hens fed a diet supplemented with a high level of CSO (4%) were noticeably increased (*p* < 0.05). Even though the supplementation of 4% CSO in diets aroused beneficial influences on liver function, a high level of CSO inclusion in laying hens’ diets is not recommended due to its hypercholesterolemia effect. In conclusion, supplementation of CSO, which contains 0.20% CPFAs, was the primary cause of alteration in fatty acid composition and cholesterol content in hens, while no interaction between CSM and CSO nor CSM effect was found for lipid profile and liver health in laying hen.

## 1. Introduction

With the rapidly increasing demand of the feed market for protein, cottonseed, as a low-cost alternative protein source of soybean, has been considered for use in feedstuff for a long time [1]. It has enjoyed widespread use in ruminant diets for decades due to the good tolerance of ruminants to the hazardous substances presented in cottonseed, namely cyclopropene fatty acids (CPFAs) and free gossypol (FG) [2]. Furthermore, cottonseed meal (CSM), a cottonseed by-product, has been widely supplemented in poultry diets in spite of its substantially lower energy and protein. Thanks to low requirements for energy and protein of laying hens, CSM is a more suitable feedstuff applied to laying hen diets than to broiler diets [3]. However, laying hens appear to be more susceptible to the CPFAs and FG present in cottonseed.

Cottonseed oil (CSO), extracted from the seed, is one of the most commonly used vegetable oils in human and animal diets. CPFAs, the micro components present in CSO, are a class of fatty acid compounds containing a cyclopropene group, commonly referred to as malvalic and sterculic acids. It was noticed very early that CPFAs had adverse biological effects in laying hens [4]. The most common hazard of them is enhancing the red discoloration of egg whites. In addition, cottonseed oil and *Sterculia foetida* seed oil, which both contain CPFAs, were reported to interfere with fatty acid metabolism in hens and change fatty acid distribution in eggs [5].

Free gossypol, a toxic phenolic pigment in cottonseed, causes numerous adverse health effects in animals, as well [6,7]. Several studies have reported that the concentrations of CPFAs and FG are the main causes leading to bad egg quality, especially of the yolk [8]. In addition to the discoloration in the yolk, the recent histological analyses of chicken livers showed substantial lipidosis and, thus, hepatic malfunction in chickens fed a high dose of gossypol [9]. Previous research reported that egg discoloration happened when laying hens were fed a diet supplemented with CSM produced by screwpress processing techniques which result in substantially more oil residue in the CSM than modern processing techniques [10]. The high level of oil residue in the CSM would lead to increased intake of CPFAs in animals. Meanwhile, in previous trials conducted in our laboratory, it was also found that either high levels of CSM or CSO inclusion in diets had adverse effects on the laying performance of hens and the fatty acid profile of their eggs [11,12]. These adverse effects were mostly all linked to the alteration of lipid metabolism in the hen liver.

However, it is not clear whether CPFAs and FG, individually or both of them, are involved in the metabolic dysfunction of lipids. The underlying causes of these adverse biological effects are unknown. If the disturbance of physiological homeostasis is caused by CPFAs or FG present in cottonseed, it should raise public safety concerns about the use of cottonseed and its by-products because of their long tradition of use in food processing [13]. Therefore, the objectives of the current study were to clarify the effects of degossypolized CSO (containing CPFAs) and CSM (containing FG) or their interaction on the fatty acid profile, lipid content in tissues and serum, and liver health of laying hens. Furthermore, this study intended to provide more insight into the application of cottonseed and its by-products in animal nutrition.

## 2. Materials and Methods

### 2.1. Animals, Design, and Experimental Diets

A total of 162 24-week-old Hy-Line Brown laying hens were allocated to nine dietary treatments in a completely randomized design using a 3 × 3 factorial arrangement with six replicates per group, and three birds in each replicate. The experimental diets were formulated based on corn–soybean-meal-based diets supplemented with 0%, 2%, or 4% CSO and 0%, 6%, or 12% CSM in place of soybean oil and soybean meal, respectively. The CSM used in this study was determined to contain 693.81 mg/kg FG and low residual oil content. The levels of free gossypol in diets supplemented with 6% and 12% CSM are 41.63 and 83.26 mg/kg, both of which exceeded the national safety limit (GB 13078-2017) of free gossypol for laying hens ration in China. The fatty acid composition of the soybean oil and the degossypolized CSO are presented in Table 1, in which CSO contained 0.20% CPFAs, composed of malvalic acid and sterculic acid, which is much higher than previously reported values for CPFAs in refined cottonseed oil [14]. The birds were reared in wire cages, and the management was consistent with the recommendations of a commercial management guide. The diets were formulated to meet the nutrient and energy requirements of laying hens (NRC, 1994) [15], and fed to hens ad libitum daily. The diet composition and nutrient contents are listed in Table 2. The fatty acid composition of experimental diets was determined after mixing (Table 3). This feeding experiment lasted for eight weeks.

After eight weeks on the experimental rations, one bird was randomly selected from each replicate. After the chickens fasted for 12 h, blood samples were collected from the wing vein. Then the serum was separated and stored at −80 °C for analysis of serum antioxidant enzyme activity, serum biochemicals, and serum lipids. After taking blood samples, birds were then sacrificed. Liver, breast muscle, leg muscle, and abdominal subcutaneous adipose of hens were collected for lipid analysis.

### 2.2. Fatty Acid Analysis

Total lipids in the tissues, including liver, breast muscle, leg muscle, and abdominal subcutaneous adipose, were extracted using chloroform–methanol, as reported by Folch et al. [16]. An improved method [11] used in our laboratory for fatty acid analysis was modified from Slover and Lanza [17]. Briefly, a gas chromatograph (GC) fitted with an SP 2560 fused silica capillary column (100 m × 0.25 mm ID, 0.20 μm film; Supelco, Bellefonte, PA, USA) was used for separating and quantifying the fatty acid methyl esters (FAMEs). The initial temperature of the oven was set at 140 °C and kept for 5 min; the oven temperature was then increased to 240 °C at a rate of 4 °C/min. Both the injector and the flame ionization detector temperature were 260 °C. The carrier gas was nitrogen at 3.0 mL/min with a split ratio of 100:1 (*v*/*v*). The peak of individual fatty acid was identified and quantified by comparison of the retention time and peak area with the standard FAME. The composition of the fatty acids is expressed as percentages of total fatty acids.

### 2.3. Determination of Cholesterol and Total Fat

Fat content in tissues, including liver, breast muscle, and leg muscle, was determined by the ethyl ether extraction method after drying. The cholesterol content in tissues was determined through a GC using a method modified from those of Botsoglou et al. [18] and Zhang et al. [19]. Three grams of fresh tissues were homogenized with 5 mL of KOH (potassium hydroxide) solution (0.5 M) in tubes and heated at 80 °C in the water bath for 20 min. Then, the tubes were removed from the water bath and cooled at ambient temperature. The extraction of cholesterol was carried out using 5 mL of hexane along with 1 mL water after drying under nitrogen. The extract was redissolved in 1 mL of pure ethanol. A 1 μL aliquot from the redissolution was injected into a gas chromatographic for cholesterol analysis fitted an Rtx-1 capillary column (30 m × 0.25 mm ID, 0.25 μm; RESTEK, Bellefonte, PA, USA). The initial temperature of the oven was 200 °C. The oven temperature was then increased to 260 °C at a rate of 10 °C/min and held there for 12 min. The injector temperature was 280 °C, whereas the flame ionization detector temperature was set at 260 °C. The flow rate of carrier gas nitrogen was set at 1 mL/min with a split ratio of 20:1 (*v*/*v*).

### 2.4. Assay of Serum Antioxidant Enzyme Activity and Serum Biochemicals

Antioxidant indices, including the activity of superoxide dismutase (SOD), glutathione peroxide (GSH-PX), and catalase (CAT), and the contents of glutathione (GSH), total antioxidant capacity (T-AOC), and malondialdehyde (MDA) in serum were measured using colorimetric methods with the commercial kits (Nanjing Jiancheng Bioengineering Institute, Nanjing, China) following the manufacturer’s instructions.

The concentration of alanine aminotransferase (ALT), aspartate aminotransferase (AST), alkaline phosphatase (ALP), total bilirubin (TBIL), glucose (GLU), total protein (TP), albumin (ALB), total cholesterol (TC), triglycerides (TGs), and low-density lipoprotein cholesterol (LDL-c) in serum were determined using an autoanalyzer (Synchron CX4 PRO, Beckman, CA, USA) with the commercial kits (Dia Sys Diagnostic System, Shanghai, China).

### 2.5. Statistical Analysis

The experimental data were analyzed by two-way variance of general linear model procedure in SPSS version 19.0. (SPSS, Inc., Chicago, IL, USA) with the levels of CSM and CSO substitution and their interaction included as main factors. Results are presented as means with SEM and Duncan’s Multiple Range Test was used for post hoc comparison.

## 3. Results

### 3.1. Fatty Acid Analysis

All fatty acids determined in hens’ livers, breast muscles, thigh muscles, and abdominal subcutaneous adipose tissues are presented in Table 4 and Supplementary Tables S1–S3, respectively. An overall increase in saturated fatty acids (SFAs) and an overall significant decrease (*p* < 0.05) in monounsaturated fatty acids (MUFAs) in the total liver lipids of hens fed a diet supplemented with CSO were observed. Liver lipids isolated from hens fed 4% CSO in the diet contained significantly higher (*p* < 0.05) levels of myristic acid (C14:0) and stearic acid (C18:0) (0.55% and 16.51%, respectively) than those from hens fed a diet without CSO (0.33% and 14.35%, respectively), but less (*p* < 0.05) oleic acid (C18:1n9). Compared with hens fed a diet without CSO, docosahexaenoic acid (DHA), an n-3 polyunsaturated fatty acid, was notability decreased (*p* < 0.05) in the liver lipids of hens fed a diet containing either 2% or 4% CSO. Additionally, the supplementation of 6% or 12% CSM to the basic diet made no difference in the fatty acid composition of the liver, except for heptadecanoic acid (C17:0). Meanwhile, a similar phenomenon of up-regulation of lipid saturation and down-regulation of lipid unsaturation also arose in breast muscle and abdominal fat, but not in the thigh muscle of these hens. Furthermore, there was no interaction (*p* > 0.05) between CSO and CSM observed on the fatty acid composition of liver lipids.

### 3.2. Cholesterol and Total Fat Analysis

Compared with hens fed a diet either with 2% CSO or without CSO, the cholesterol content in the leg muscles of hens fed a diet supplemented with a high level of CSO (4%) was significantly increased (*p* < 0.05, Table 5). Feeding with a diet supplemented with CSO also markedly increased (*p* < 0.05) the cholesterol content in hens’ breast muscles, while CSO supplementation did not alter liver cholesterol content. Unlike cholesterol, the total fat in tissues was not responsive to dietary manipulation. Results showed that the levels of total fat in the liver, breast muscle, and leg muscle were not influenced by dietary CSO or CSM (Table 5).

### 3.3. Serum Lipid Biochemistry and Antioxidant Variables

The part of serum lipid variables involved in lipid metabolism was significantly affected by supplementation with CSO, but not with CSM (Table 6). Compared to hens fed a diet without CSO, the serum concentrations of TC and LDL-c of hens fed a diet supplemented with a high level of CSO (4%) were significantly increased (*p* < 0.05), while these variables were not notably different (*p* > 0.05) in the low-level CSO (2%) group from those of hens fed a diet without CSO. Meanwhile, some serum biochemical parameters, such as ALT, TP, and ALB, were changed by CSO inclusion (Table 7). The level of ALT in hens’ serum was dropped by feeding CSO, while the TP and ALB were markedly increased by feeding a high level (4%) of CSO (*p* < 0.05).

The effects of dietary CSO and CSM supplementation, individually or in combination, on serum antioxidant variables are presented in Table 8. As shown in the results, CSO was the only factor affecting the antioxidant capacity in hens’ serum. Feeding a diet with a high level (4%) of CSO resulted in an increase of T-AOC and a decrease of GSH-PX in hens’ serum (*p* < 0.05). A noticeable decrease (*p* < 0.05) of MDA was observed in the serum of hens fed a diet supplemented with either 2% or 4% CSO.

## 4. Discussion

The changes in the fatty acid composition of hens’ tissue among different groups indicate that CSO, rather than CSM, dramatically altered the fatty acid profile of laying hens. No interaction between CSO and CSM was found for fatty acid composition. There are at least two possible causes contributing to the alteration of fatty acid composition caused by CSO. First, the fatty acid composition in hens’ tissues is susceptible to changes of the fatty acids taken in from dietary fat, which is usually included in the hen diet as part of the source of energy [20]. Alteration in dietary lipid intake has profound influences on the fatty acid profiles of tissues. As shown in a previous study, laying hens had much more n-3 polyunsaturated fatty acid (PUFA) build-up in thighs, breasts, and egg yolks when fed a hemp oil diet enriched in n-3 PUFAs [21]. The feature of the CSO used in the current study is that had a remarkably higher level of palmitic acid (C16:0) and a significantly lower level of ALA (alpha-linolenic acid, C18:3n3) than those of the soybean oil (Table 1); consequently, that storage of fat in adipose tissue, such as abdominal fat, breast fat, and leg fat from hens fed a CSO diet, containing more palmitic acid and less ALA could be attributed to the fatty acid properties of the CSO to some extent. Considering that DHA does not occur in CSO, its decreasing concentration of tissue DHA in response to the level of CSO incorporation in diets could be due to the lesser amount of ALA initially present in CSO compared with that in soybean oil. ALA has been reported as a precursor for the in vivo synthesis of these n-3 fatty acids in hen livers [22].

Second, there were some special fatty acids—CPFAs, commonly referred to as malvalic (C18:1 cpe) and sterculic acids (C19:1 cpe)—present in CSO, probably interfering with de novo synthesis of fatty acids. Their presence in CSO has caused many deleterious biological effects when fed to animals [23,24]. The most attractive biological property of CPFAs is supposed to be the potent inhibition of stearoyl-CoA desaturase (SCD) [25]. SCD is a membrane-bound delta-9 desaturase catalyzing the insertion of a double bond into the ninth carbon of saturated C16 and C18 substrates, thereby converting them to MUFAs [26]. In the research reported here, the CSO added to hens’ diets contained 0.12% malvalic acid and 0.08% sterculic acid, and 0.20% CPFAs in total (Table 1). From the results on fatty acid composition in abdominal subcutaneous adipose and breast muscle (Tables S1 and S2), we found the level of C18:0 in tissues was significantly increased by feeding hens CSO diets even though the ratio of C18:0 in CSO is much lower than that in soybean oil. That is an indication the CPFAs in CSO inhibited the conversion of C18:0 to its delta-9 monounsaturated counterparts, C18:1 in hen liver.

In addition, there are also other factors that affect the fatty acid composition of fat from different tissue. Commonly, adipose fatty acid composition is considered to reflect dietary intake, but it is not an exact mirror, suggesting the requirement of de novo biosynthesis of these fatty acids in addition to the dietary sources [27]. It has already been proven that increased fat intake from diets could efficiently depress the de novo biosynthesis of lipid in animals [28,29] and specific dietary fatty acids, such as C16:0, also decrease the rate of de novo biosynthesis of lipid [30]. In the present study, C16:0 comprised 19.8% in the CSO, which was more than twice the ratio of C16:0 in the soybean oil.

In addition to the alteration of fatty acid profile in tissues, hens fed a diet supplemented with a high level (4%) of CSO also markedly increased the cholesterol content in hens’ breast and leg muscles, as well as serum cholesterol content. According to the studies with regard to lipid metabolism of laying hens, it was known that most of the cholesterol deposited in the egg is from the liver synthesis and transported to yolk via apolipoprotein [31]. In data from our previous research [11], it can be found that a diet containing 4% CSO markedly reduces the content of cholesterol in the yolk, by 6.1%. Therefore, we speculated that the decrease of the cholesterol storage in yolk resulted in an increase in plasma TC and LDL-c of hens. The reason why transportation of the cholesterol from plasma to egg yolk was impaired was probably the reduced activities of hepatic cholesterol esterase and plasma lecithin-cholesterol acyltransferase (LCAT) caused by those excessive saturated serum lipids [32]. Moreover, the preponderance of evidence continued to indicate that serum cholesterol highly responds to saturated fatty acids in diets [33]. A literature survey concluded that serum cholesterol concentration is directly related to the degree of atherosclerosis in poultry, which suggested a long-term high level of serum cholesterol is bad for cardiovascular health [34].

According to results from the effects of CSO and CSM on serum biochemistry and antioxidant variables, it could be concluded that the high level (4%) of CSO had a beneficial influence on liver function. The level of ALT in hens’ serum was dropped by feeding CSO, while the TP and ALB were markedly increased, which commonly indicates improved hepatic function [35]. However, the data about performances of the laying hens (Table S4) had been published in our previous research [12], and it showed that a high level of CSO inclusion impaired the laying hen performances, such as decreased egg production, reduced egg weight, and low feed conversion rate. A previous study also reported the growth retardation and moderate liver histological damage of male New Zealand rabbits fed a diet containing CPFAs [36]. It was proven that CPFAs could affect animal performances through liver damage. The discrepancy in these studies might be attributed to either a comparatively low concentration of CPFAs in experimental diets used in the present study. Noticeably, MDA, a lipid peroxidation marker, was notably decreased in hen serum from CSO groups, which was generated from a process under which oxidants such as free radicals attack lipids containing carbon–carbon double bonds, especially PUFAs [37]. It was likely that MDA decreased in response to the down-regulation of lipid unsaturation.

There was almost no interaction between CSO and CSM nor the CSM effect found for lipid profile and hepatic function in laying hens. That was inconsistent with Blevins et al. [9], who reported high gamma glutamyltransferase (GGT) and AST, indicators of liver function, in chickens fed diets containing gossypol, with high lipidosis scores observed from histological measures. The relatively lower dose of gossypol (83.25 mg/kg) and different dietary manipulation in the present study might be the reasons why no CSM effect was found for lipid content and liver function, compared to former research in which gossypol was added as pure material at a dose of 1000 mg/kg.

## 5. Conclusions

In conclusion, the present study showed that dietary supplementation of CSO, containing 0.20% CPFAs, is the major factor leading to alteration in lipid profiles in hens, which contributes to the down-regulation of fatty acid unsaturation in fat and up-regulation in the level of cholesterol in tissues as well as serum. Meanwhile, CSM hardly interacted with CSO on lipids in laying hens. Probable reasons involved in the changes of lipid profiles caused by dietary CSO inclusion are (1) higher fatty acid saturation of dietary fat, CSO, when compared with soybean oil and (2) CPFAs, potent inhibitors of SCD, present in CSO. Therefore, a high level (4%) of CSO inclusion in laying hens’ diets is not recommended due to its hypercholesterolemia effect. Taken together, more consideration should be given when supplementing CSO or cottonseed by-products with a high residual oil level into the diet as sources of nutrients. It should raise public safety concerns about the application of CSO because of its long tradition of use in human food processing.

## Figures and Tables

**Table 1:** Fatty acid composition of soybean oil and cottonseed oil (g/100 g of total fatty acids).

Fatty Acid	Soybean Oil	Cottonseed Oil
C16:0	8.05	19.78
C17:0	0.05	0.06
C18:0	2.84	1.57
C20:0	2.52	1.25
C22:0	0.20	0.15
C24:0	0.07	0.05
C16:1	0.05	0.40
C18:1n9*t*	0.20	0.02
C18:1n9*c*	17.45	15.59
C18:2n6*c*	52.23	54.50
C18:3n6	0.43	0.17
C18:3n3	8.09	0.75
C20:2	0.30	0.18
C18:1 cpe ^1^	ND ^2^	0.12
C19:1 cpe	ND	0.08

^1^ C18:1 cpe, malvalic acid; C19:1 cpe, sterculic acid. ^2^ ND, not detected.

**Table 2:** Diets composition and nutrient contents.

Items	0% CSO ^1^			2% CSO			4% CSO		
	0% M ^2^	6% M	12% M	0% M	6% M	12% M	0% M	6% M	12% M
Ingredient	%	%	%	%	%	%	%	%	%
Corn	52.2	52.2	52.2	52.2	52.2	52.2	52.2	52.2	52.2
Wheat bran	6.00	6.00	6.00	6.00	6.00	6.00	6.00	6.00	6.00
Soybean meal	26.1	20.1	14.1	26.1	20.1	14.1	26.1	20.1	14.1
Cottonseed meal ^3^	–	6.00	12.0	–	6.00	12.0	–	6.00	12.0
Cottonseed oil	–	–	–	2.00	2.00	2.00	4.00	4.00	4.00
Soybean oil	4.00	4.00	4.00	2.00	2.00	2.00	0.00	0.00	0.00
Dicalcium phosphate	1.53	1.38	1.22	1.53	1.38	1.22	1.53	1.38	1.22
Limestone	8.76	8.85	8.96	8.76	8.85	8.96	8.76	8.85	8.96
Salt	0.30	0.30	0.30	0.30	0.30	0.30	0.30	0.30	0.30
Lysine	0.00	0.06	0.11	0.00	0.06	0.11	0.00	0.06	0.11
Methionine	0.11	0.11	0.11	0.11	0.11	0.11	0.11	0.11	0.11
Premix ^4^	1.00	1.00	1.00	1.00	1.00	1.00	1.00	1.00	1.00
Calculated nutrient level									
Metabolizable energy MJ/kg	11.45	11.36	11.27	11.51	11.42	11.33	11.57	11.47	11.38
Crude protein	16.6	16.6	16.6	16.6	16.6	16.6	16.6	16.6	16.6
Calcium	3.51	3.50	3.50	3.51	3.50	3.50	3.51	3.50	3.50
Total phosphorus	0.61	0.61	0.61	0.61	0.61	0.61	0.61	0.61	0.61
Available phosphorus	0.38	0.37	0.35	0.38	0.37	0.35	0.38	0.37	0.35
Lysine	0.85	0.86	0.85	0.85	0.86	0.85	0.85	0.86	0.85
Methionine	0.35	0.35	0.35	0.35	0.35	0.35	0.35	0.35	0.35

^1^ CSO, cottonseed oil. ^2^ M, cottonseed meal (CSM). ^3^ Residual oil content of 0.8%. ^4^ Provided the following (per kg of diet): vitamin A, 12,000 IU; vitamin D3, 4000 IU; vitamin E, 35 IU; vitamin K, 5 mg; thiamine, 2 mg; riboflavin, 8 mg; pyridoxine, 5 mg; vitamin B12, 50 μg; D-biotin, 200 μg; pantothenic acid, 15 mg; nicotinic acid, 50 mg; choline, 500 mg; folic acid, 1.5 mg; Mn, 120 mg; Zn, 80 mg; Fe, 120 mg; Cu, 15 mg; I, 1 mg; Se, 0.3 mg.

**Table 3:** Fatty acid composition of experimental diets (g/100g of total fatty acids).

Fatty Acids	0% CSO			2% CSO			4% CSO		
	0% M ^2^	6% M	12% M	0% M	6% M	12%M	0% M	6% M	12% M
C16:0	10.01	10.20	10.18	13.64	13.95	14.12	17.76	17.67	17.93
C18:0	3.07	3.04	3.03	2.66	2.65	2.61	2.18	2.22	2.13
C20:0	2.06	2.01	2.03	1.57	1.59	1.56	1.18	1.16	1.13
C22:0	0.15	0.15	0.15	0.13	0.13	0.13	0.12	0.11	0.11
C16:1	0.10	0.10	0.10	0.21	0.22	0.22	0.33	0.33	0.34
C18:1n9t	0.15	0.15	0.14	0.09	0.09	0.08	–	–	–
C18:1n9c	22.57	22.87	22.48	22.02	21.96	21.93	21.01	21.21	21.12
C18:2n6c	54.92	54.77	54.96	55.39	55.26	55.27	55.65	55.69	55.77
C18:3n6	0.32	0.32	0.31	0.24	0.22	0.22	0.15	0.14	0.14
C18:3n3	6.42	6.17	6.38	3.87	3.74	3.67	1.43	1.33	1.19
C20:2	0.22	0.22	0.23	0.18	0.18	0.18	0.14	0.13	0.14

**Table 4:** Effects of cottonseed oil (CSO) and cottonseed meal (CSM) on fatty acid composition (g/100 g of total fatty acids) of livers in laying hens ^1^.

Items	Main Effects							*p*-Value		
	CSO, %			CSM, %						
	0	2	4	0	6	12	SEM	CSO	CSM	O × M
C14:0	0.33 ^b^	0.37 ^b^	0.55 ^a^	0.43	0.44	0.39	0.025	<0.001	0.23	0.55
C16:0	23.68	23.03	23.29	23.14	23.47	23.39	0.151	0.38	0.71	0.65
C16:1	0.71	0.74	0.80	0.80	0.73	0.73	0.041	0.65	0.80	0.26
C17:0	0.48	0.41	0.39	0.53 ^a^	0.37 ^b^	0.39 ^b^	0.026	0.24	0.04	0.48
C18:0	14.35 ^b^	16.09 ^ab^	16.51 ^a^	16.25	15.01	15.72	0.370	0.049	0.34	0.99
C18:1	24.25 ^a^	25.1 ^a^	21.76 ^b^	23.45	23.70	23.95	0.498	0.03	0.89	0.32
C18:2	24.72	27.51	26.85	25.70	27.24	26.15	0.442	0.09	0.27	0.87
C18:3n3	0.90	0.84	0.73	0.88	0.77	0.82	0.036	0.14	0.76	0.78
C18:3n6	0.51	0.65	0.64	0.68	0.63	0.49	0.050	0.58	0.32	0.12
C20:2	0.6 ^b^	0.7 ^ab^	0.85 ^a^	0.76	0.60	0.79	0.040	0.03	0.11	0.14
C20:3n6	0.56	0.42	0.49	0.40	0.49	0.58	0.036	0.43	0.20	0.94
C20:4n6	5.79 ^a^	3.66 ^b^	5.54 ^a^	4.82	4.91	5.25	0.306	0.006	0.68	0.05
C22:6n3	2.58 ^a^	1.32 ^b^	1.59 ^b^	1.90	1.64	1.94	0.142	0.001	0.33	0.37
SFAs	38.85	39.93	40.75	40.40	39.29	39.89	0.600	0.15	0.55	0.99
MUFAs	25.23 ^a^	25.84 ^a^	22.55 ^b^	24.24	24.64	24.68	0.518	0.043	0.91	0.42
PUFAs	35.66	35.10	36.69	35.15	36.29	36.02	0.440	0.34	0.47	0.56
n-3 PUFAs	3.48 ^a^	2.16 ^b^	2.32 ^b^	2.79	2.41	2.76	0.147	<0.001	0.26	0.19
n-6 PUFAs	31.57	32.24	33.53	31.60	33.28	32.47	0.417	0.26	0.26	0.83
n-3/n-6	0.11 ^a^	0.07 ^b^	0.07 ^b^	0.09	0.08	0.09	0.005	<0.001	0.09	0.23

^1^ Data are expressed as means (*n* = 18) and SEM is the standard error of the means. ^a,b^ Mean values with different letters are significantly different (*p* < 0.05). Saturated fatty acids, monounsaturated fatty acids, and polyunsaturated fatty acids are expressed as SFAs, MUFAs, and PUFAs, respectively.

**Table 5:** Effects of cottonseed oil (CSO) and cottonseed meal (CSM) on cholesterol and fat content of different tissues in laying hens ^1^.

CSO	CSM	Cholesterol			Total Fat		
		Breast Muscle	Leg Muscle	Liver	Liver	Breast Muscle	Leg Muscle
%	%	mg/100 g	mg/100 g	mg/100 g	g/100 g	g/100 g	g/100 g
	0	48.44	49.03	200.15	9.38	2.63	5.11
0	6	36.08	48.94	208.87	9.33	3.78	5.07
	12	42.04	52.81	195.83	9.25	2.57	5.15
	0	33.70	54.92	182.71	9.31	3.63	5.12
2	6	43.28	60.03	183.83	9.36	2.75	4.85
	12	51.40	55.73	209.04	9.27	2.46	4.93
	0	52.45	52.94	187.25	9.34	2.74	5.19
4	6	59.72	56.36	190.32	9.35	2.50	5.06
	12	62.17	60.13	197.62	9.34	3.05	5.13
SEM		1.934	1.177	2.935	0.441	0.151	0.050
Main effects							
CSO, %							
0		42.18 ^b^	50.26 ^b^	202.34	9.32	3.04	5.11
2		42.80 ^b^	56.89 ^a^	191.36	9.31	2.93	4.96
4		57.82 ^a^	56.21 ^a^	191.73	9.34	2.76	5.13
CSM, %							
0		44.17	52.30	190.47	9.34	3.00	5.14
6		46.17	55.11	194.34	9.34	3.01	4.99
12		50.58	55.94	200.95	9.28	2.72	5.07
*p*-value							
CSO		<0.001	0.04	0.30	0.99	0.80	0.39
CSM		0.09	0.36	0.34	0.98	0.63	0.51
O × M		0.02	0.70	0.31	1.00	0.10	0.95

^1^ Data are expressed as means (*n* = 6) and SEM is the standard error of the means. ^a,b^ Mean values with different letters are significantly different (*p* < 0.05).

**Table 6:** Effects of cottonseed oil (CSO) and cottonseed meal (CSM) on serum lipids in laying hens ^1^.

CSO	CSM	TC	TGs	LDL-c
%	%	mmol/L	mmol/L	mmol/L
	0	1.71	5.67	0.32
0	6	1.35	4.20	0.42
	12	1.75	6.28	0.46
	0	1.71	6.92	0.58
2	6	1.73	4.77	0.45
	12	1.75	4.97	0.52
	0	2.26	5.70	0.53
4	6	2.65	6.31	0.96
	12	2.01	6.14	0.94
SEM		0.093	0.457	0.069
Main Effects				
CSO, %				
0		1.60 ^b^	5.38	0.40 ^b^
2		1.73 ^b^	5.55	0.52 ^b^
4		2.31 ^a^	6.05	0.80 ^a^
CSM, %				
0		1.90	6.10	0.48
6		1.91	5.09	0.61
12		1.84	5.79	0.64
*p*-value				
CSO		0.004	0.21	<0.001
CSM		0.94	0.17	0.11
O × M		0.35	0.71	0.06

^1^ Data are expressed as means (*n* = 6) and SEM is the standard error of the means. ^a,b^ Mean values with different letters are significantly different (*p* < 0.05). Total cholesterol, TC; triglycerides, TGs; low-density lipoprotein cholesterol, LDL-c.

**Table 7:** Effects of cottonseed oil (CSO) and cottonseed meal (CSM) on serum biochemical parameters in laying hens ^1^.

CSO	CSM	ALT	AST	ALP	TP	ALB	TBIL	GLU
%	%	U/L	U/L	U/L	g/L	g/L	nmol/L	mmol/L
	0	2.83	252.2	655.3	39.0	17.4	3.75	11.54
0	6	2.88	211.2	566.3	30.0	15.1	3.48	10.28
	12	2.60	321.7	831.0	31.7	14.9	3.70	10.88
	0	2.25	317.1	618.9	34.1	16.2	3.67	10.97
2	6	1.97	248.3	567.1	37.1	16.9	3.82	10.95
	12	1.75	214.0	621.6	36.9	17.8	3.80	11.20
	0	2.20	248.2	1031.6	46.3	18.7	3.55	11.43
4	6	2.20	218.9	845.8	42.1	18.1	3.77	11.84
	12	1.97	248.7	653.6	45.2	20.1	3.82	12.71
SEM		0.100	13.32	50.92	1.01	0.29	0.030	0.175
Main Effects								
CSO, %								
0		2.77 ^a^	261.7	684.2	33.60 ^b^	15.80 ^b^	3.64	10.90 ^b^
2		1.99 ^b^	259.8	602.5	36.00 ^b^	17.00 ^b^	3.76	11.04 ^b^
4		2.12 ^b^	238.6	843.7	44.50 ^a^	19.00 ^a^	3.71	11.99 ^a^
CSM, %								
0		2.43	272.5	768.6	39.80	17.40	3.66	11.32
6		2.35	226.1	659.7	36.40	16.70	3.69	11.02
12		2.11	261.5	702.7	37.90	17.60	3.78	11.59
*p*-value								
CSO		0.003	0.79	0.15	<0.001	<0.001	0.24	0.02
CSM		0.35	0.43	0.68	0.19	0.13	0.22	0.37
O × M		0.97	0.37	0.42	0.09	0.01	0.05	0.31

^1^ Data are expressed as means (*n* = 6) and SEM is the standard error of the means. ^a,b^ Mean values with different letters are significantly different (*p* < 0.05). Alanine aminotransferase, ALT; aspartate aminotransferase, AST; alkaline phosphatase, ALP; total protein, TP; albumin, ALB; total bilirubin, TBIL; glucose, GLU.

**Table 8:** Effects of cottonseed oil (CSO) and cottonseed meal (CSM) on serum antioxidant parameters in laying hens ^1^.

CSO	CSM	T-AOC	SOD	CAT	GSH-PX	GSH	MDA
%	%	mM	U/mL	U/mL	Activity Unit	g/L	g/L
	0	0.73	94.01	6.99	2014.61	2.57	5.97
0	6	0.71	95.82	6.98	2672.70	3.87	5.28
	12	0.64	97.48	4.33	2763.13	2.65	6.39
	0	0.62	96.92	6.33	2438.03	3.17	4.10
2	6	0.77	95.42	6.23	2461.22	3.01	4.38
	12	0.76	95.26	6.24	3005.22	4.53	5.26
	0	0.61	101.29	6.26	2220.52	1.78	3.67
4	6	0.76	99.72	6.32	1967.31	4.60	4.94
	12	0.71	99.67	6.64	2117.01	4.06	5.08
SEM		0.024	0.638	0.181	81.623	0.303	0.23
Main Effects							
CSO, %							
0		0.70	95.86 ^b^	6.10	2448.52 ^a^	2.98	5.87 ^a^
2		0.72	95.83 ^b^	6.27	2659.42 ^a^	3.59	4.61 ^b^
4		0.70	100.22 ^a^	6.41	2093.22 ^b^	3.48	4.57 ^b^
CSM, %							
0		0.66	97.65	6.52	2196.16	2.47	4.52
6		0.75	96.98	6.51	2367.07	3.82	4.87
12		0.70	97.60	5.71	2644.59	3.75	5.57
*p*-value							
CSO		0.91	0.004	0.74	0.01	0.74	0.02
CSM		0.29	0.94	0.09	0.09	0.15	0.16
O × M		0.53	0.62	0.01	0.13	0.34	0.67

^1^ Data are expressed as means (*n* = 6) and SEM is the standard error of the means. ^a,b^ Mean values with different letters are significantly different (*p* < 0.05). Total antioxidant capacity, T-AOC; superoxide dismutase, SOD; catalase, CAT; glutathione peroxide, GSH-PX; glutathione, GSH; malondialdehyde, MDA.

## Data Availability

The data presented in this study are available on fair request from the respective author.

## Supplementary Materials

The following are available online at https://www.mdpi.com/2076-2615/11/1/78/s1. Table S1: Effects of cottonseed oil (CSO) and cottonseed meal (CSM) on fatty acid composition (g/100 g of total fatty acids) of abdominal subcutaneous adipose tissue in laying hens, Table S2: Effects of cottonseed oil (CSO) and cottonseed meal (CSM) on fatty acid composition (g/100 g of total fatty acids) of breast muscle in laying hens, Table S3: Effects of cottonseed oil (CSO) and cottonseed meal (CSM) on fatty acid composition (g/100 g of total fatty acids) of leg muscle in laying hens, Table S4: Effects of cottonseed oil (CSO) and cottonseed meal (CSM) on laying performance.
