# Supplementary material for: Effects of Dietary Cottonseed Oil and Cottonseed Meal Supplementation on Liver Lipid Content, Fatty Acid Profile and Hepatic Function in Laying Hens
Source: Animals (Basel). 2021 Jan 4;11(1):78. doi: 10.3390/ani11010078 (PMC7824706; doi:10.3390/ani11010078)
Supplement: Supplementary file 1 [file animals-11-00078-s001.pdf]

animals

Article

Effects of Dietary Cottonseed Oil and Cottonseed Meal Supplementation on Liver Lipid Content, Fatty Acid Profile and Hepatic Function in Laying Hens

Ao Yang, Cong Zhang, Beiyu Zhang, Zhiyun Wang, Luoyi Zhu, Yang Mu, Shuai Wang, and Desheng Qi *

Department of Animal Nutrition and Feed Science, College of Animal Science and Technology, Huazhong Agricultural University, Wuhan, Hubei 430070, China; yangao@webmail.hzau.edu.cn (A.Y.); zhangcong@webmail.hzau.edu.cn (C.Z.); zhangbeiyu@webmail.hzau.edu.cn (B.Z.); zhiyun_wang_hzau@163.com (Z.W.); 11917023@zju.edu.cn (L.Z.); 20140017@hbut.edu.cn (Y.M.); wangshuai@mail.hzau.edu.cn (S.W.)

* Correspondence: qds@mail.hzau.edu.cn

Citation: Yang, A.; Zhang, C.; Zhang, B.; Wang, Z.; Zhu, L.; Mu, Y.; Wang, S.; Qi, D. Effects of Dietary Cottonseed Oil and Cottonseed Meal Supplementation on Liver Lipid Content, Fatty Acid Profile and Hepatic Function in Laying Hens. *Animals* **2021**, *11*, 78. http://doi.org/10.3390/ ani11010078

Received: 27 November 2020
Accepted: 30 December 2020
Published: 04 January 2021

Publisher's Note: MDPI stays neutral with regard to jurisdictional claims in published maps and institutional affiliations.

Copyright: © 2020 by the authors. Submitted for possible open access publication under the terms and conditions of the Creative Commons Attribution (CC BY) license (http://creativecommons.org/licenses/by/4.0/).

Supplementary materials:

Table S1. Effects of cottonseed oil (CSO) and cottonseed meal (CSM) on fatty acid composition (g/100 g of total fatty acids) of abdominal subcutaneous adipose tissue in laying hens[1].

	Main effects									
	**CSO, %**			CSM, %				*P*-value		
**Items**	**0**	**2**	**4**	0	6	12	SEM	CSO	CSM	O×M
C14:0	0.33c	0.44b	0.51a	0.42	0.43	0.43	0.012	<0.001	0.40	0.78
C14:1	0.09b	0.11a	0.11a	0.10	0.10	0.11	0.003	<0.001	0.06	0.27
C16:0	18.03c	19.52b	21.32a	19.36	19.98	19.53	0.223	<0.001	0.08	0.52
C16:1	1.49	1.31	1.50	1.34	1.48	1.49	0.045	0.20	0.27	0.11
C17:0	0.13c	0.22a	0.19b	0.2a	0.16ab	0.18b	0.008	<0.001	0.00	0.00
C17:1	0.19b	0.21a	0.18b	0.20	0.19	0.19	0.005	0.02	0.09	0.44
C18:0	5.47c	7.06b	7.51a	6.79	6.62	6.59	0.153	<0.001	0.67	0.31
C18:1n9	36.49a	32.43b	31.04b	33.27	33.37	33.43	0.447	<0.001	0.96	0.30
C18:2n6	35.20	36.35	35.70	35.98	35.45	35.76	0.367	0.51	0.82	0.37
C18:3n6	0.18a	0.18a	0.08b	0.18	0.13	0.12	0.013	0.001	0.11	0.56
C20:1	0.23b	0.28a	0.27a	0.27	0.25	0.26	0.006	0.001	0.24	0.53
C18:3n3	1.94a	1.6b	1.33c	1.64	1.61	1.63	0.049	<0.001	0.90	0.21
C20:2	0.13c	0.19a	0.17b	0.16b	0.16b	0.18a	0.005	<0.001	0.00	0.007
C20:3n6	0.04a	0.02b	0.04a	0.04	0.03	0.03	0.003	0.04	0.33	0.15
C20:4n6	0.05	0.06	0.06	0.06	0.06	0.06	0.003	0.50	0.20	0.15
SFAs	23.97c	27.25b	29.52a	26.77	27.19	26.73	0.362	<0.001	0.55	0.81
MUFAs	38.49a	34.35b	33.1b	35.17	35.39	35.49	0.468	<0.001	0.91	0.26
PUFAs	37.55	38.41	37.38	38.07	37.43	37.79	0.389	0.56	0.79	0.46
n-3 PUFAs	1.94a	1.6b	1.33c	1.64	1.61	1.63	0.049	<0.001	0.90	0.21
n-6 PUFAs	35.48	36.60	35.88	36.27	35.67	35.97	0.369	0.52	0.79	0.38
n-3/n-6	0.06a	0.04b	0.04c	0.05	0.05	0.05	0.001	<0.001	0.43	0.16

[1]The data are expressed as means (n=18) and SEM is the standard error of the means. [a-c]means values with unlike letters were significantly different (P <0.05). Saturated fatty acids, monounsaturated fatty acids and polyunsaturated fatty acids are expressed in SFAs, MUFAs and PUFAs, respectively.

Table S2. Effects of cottonseed oil (CSO) and cottonseed meal (CSM) on fatty acid composition (g/100 g of total fatty acids) of breast muscle in laying hens[1].

	Main effects									
	**CSO, %**			CSM, %				*P*-value		
**Items**	**0**	**2**	**4**	0	6	12	SEM	CSO	CSM	O×M
C14:0	0.58	0.56	0.55	0.52	0.57	0.59	0.035	0.26	0.84	0.43
C14:1	4.16b	3.74b	5.57a	4.68	4.29	4.39	0.247	0.003	0.56	0.011
C16:0	21.81b	22.12b	22.97a	21.77b	22.67a	22.43a	0.143	<0.001	0.005	0.26
C16:1	1.47a	1.19b	1.05b	1.14	1.28	1.29	0.056	0.001	0.23	0.007
C17:0	0.80b	0.90b	1.24a	0.99	0.94	1.03	0.055	0.002	0.75	0.08
C18:0	8.61b	9.30a	9.80a	9.30	9.00	9.47	0.137	0.001	0.27	0.59
C18:1	27.96a	25.54b	22.48c	25.58	24.92	25.34	0.473	<0.001	0.53	0.003
C18:2	24.17	25.35	23.88	24.11	24.91	24.28	0.431	0.34	0.74	0.44
C18:3n3	1.50a	1.23b	0.91c	1.26	1.24	1.13	0.053	<0.001	0.50	0.62
C18:3n6	0.53	0.43	0.66	0.39	0.63	0.60	0.056	0.16	0.06	0.011
C20:2	0.50	0.42	0.55	0.49	0.50	0.47	0.031	0.07	0.85	0.016
C20:3n6	0.38b	0.37b	0.52a	0.41	0.44	0.41	0.038	0.005	0.71	0.03
C20:4n6	5.84b	5.65b	8.06a	6.78	6.35	6.40	0.316	0.002	0.76	0.047
C22:6	1.78	1.47	2.00	1.78	1.71	1.74	0.098	0.08	0.95	0.09
SFAs	31.90b	32.99b	34.47a	32.66	33.21	33.52	0.257	<0.001	0.28	0.87
MUFAs	33.45a	31.21b	29.11c	31.53	31.01	31.23	0.381	<0.001	0.75	0.06
PUFAs	34.65	35.81	36.74	35.81	35.79	35.58	0.341	0.07	0.94	0.47
n-3 PUFAs	3.28a	2.70b	2.91ab	3.04	2.95	2.87	0.093	0.04	0.73	0.29

n-6 PUFAs	30.93	30.63	31.11	28.24	32.32	32.25	0.912	0.97	0.14	0.84
n-3/n-6	0.11a	0.08b	0.08b	0.09	0.09	0.09	0.004	0.008	0.76	0.17

[1]The data are expressed as means (n =18) and SEM is the standard error of the means.[a-c]means values with unlike letters were significantly different (*P* <0.05). Saturated fatty acids, monounsaturated fatty acids and polyunsaturated fatty acids are expressed in SFAs, MUFAs and PUFAs, respectively.

Table S3. Effects of cottonseed oil (CSO) and cottonseed meal (CSM) on fatty acid composition (g/100 g of total fatty acids) of leg muscle in laying hens[1.]

	**Main effects**									
	**CSO, %**			CSM, %				*P*-value		
**Items**	**0**	**2**	4	0	6	12	SEM	CSO	CSM	O×M
C14:0	0.58	0.62	0.64	0.63	0.60	0.62	0.012	0.19	0.63	0.42
C14:1	1.65	1.67	1.42	1.27	1.85	1.58	0.111	0.57	0.06	0.69
C16:0	20.20b	20.40b	21.54a	21.24a	20.61b	20.27b	0.172	0.002	0.04	0.60
C16:1	1.92	1.69	1.86	1.87	1.76	1.85	0.055	0.23	0.74	0.95
C17:0	0.53	0.63	0.51	0.43	0.65	0.57	0.045	0.55	0.14	0.18
C18:0	9.94	10.49	10.58	10.13	10.74	10.12	0.242	0.56	0.51	0.23
C18:1	28.24	26.61	28.15	29.82	25.84	27.52	0.532	0.33	0.004	0.20
C18:2	29.54	30.36	29.69	29.42	29.52	30.57	0.275	0.46	0.15	0.10
C18:3n3	1.66a	1.56a	1.24b	1.39	1.46	1.60	0.011	<0.001	0.10	0.09
C18:3n6	0.26	0.27	0.27	0.28	0.25	0.28	0.048	0.94	0.36	0.30
C20:2	0.44	0.37	0.39	0.39	0.41	0.41	0.016	0.09	0.70	0.50
C20:3n6	0.29	0.25	0.23	0.24	0.27	0.27	0.019	0.07	0.37	0.94
C20:4n6	4.10	3.47	3.52	3.12	4.10	3.86	0.277	0.47	0.23	0.59
C22:6	0.92	0.71	0.59	0.57	0.91	0.72	0.078	0.11	0.08	0.46
SFAs	31.45	31.53	31.98	31.80	32.60	30.49	0.480	0.91	0.20	0.21
MUFAs	29.76	29.77	29.46	30.90	29.45	28.66	0.871	0.97	0.53	0.31
PUFAs	37.16	36.75	36.13	35.41b	36.63ab	38.01a	0.396	0.52	0.04	0.73
n-3 PUFAs	2.53a	2.27a	1.84b	1.96b	2.37a	2.30ab	0.086	0.00	0.05	0.70
n-6 PUFAs	2.07	8.89	1.91	8.53	2.65	2.21	0.775	0.45	0.09	0.28
n-3/n-6	0.07a	0.06ab	0.05b	0.06b	0.07a	0.07a	0.003	0.011	0.04	0.40

[1]The data are expressed as means (n = 18) and SEM is the standard error of the means. [a-c]means values with unlike letters were significantly different (*P* <0.05). Saturated fatty acids, monounsaturated fatty acids and polyunsaturated fatty acids are expressed in SFAs, MUFAs and PUFAs, respectively.

Table S4. Effects of cottonseed oil (CSO) and cottonseed meal (CSM) on laying performance[1.]

**CSO**	**CSM**	**Hen-day egg production**	**Egg weight**	**Egg mass**	**Feed per egg**
%	%	%	g	g/hen per day	g
	0	95.8	58.5	56.6	111.7
0	6	95.0	59.5	57.3	112.9
	12	95.1	58.4	56.5	114.6
	0	92.0	58.5	55.5	102.4
2	6	94.7	58.1	56.4	112.2
	12	95.3	56.6	54.6	112.8
	0	88.5	57.0	52.9	118.0
4	6	76.9	58.6	49.8	125.6
	12	95.7	57.6	56.0	102.1
SEM		0.01	0.11	0.33	0.81
Main effects					
CSO, %					
0		95.3^{b}	58.8^{b}	56.8^{b}	113.1^{ab}
2		94.0^{b}	57.7^{a}	54.5^{b}	109.2^{a}
4		87.0^{a}	57.7^{a}	52.9^{a}	115.2^{b}
CSM, %					
0		92.1^{b}	58.0^{a}	55.0	110.7^{a}
6		88.9^{a}	58.7^{b}	54.5	116.9^{b}
12		95.4^{c}	57.5^{a}	55.7	109.8^{a}
P-value					

CSO	0.00	0.00	0.00	0.00
CSM	0.00	0.00	0.14	0.00
O × M	0.00	0.00	0.00	0.00

[1]Data were cited from our previous work (Mu *et al.*, 2019).Data are expressed as means and SEM is the standard error of the means. [a–c]Mean values with different letters are significantly different ($p < 0.05$).
